# Supplementary material for: PAH-former: Transfer learning for efficient discovery of pulmonary arterial hypertension-associated genes
Source: PLoS One. 2026 Mar 6;21(3):e0344084. doi: 10.1371/journal.pone.0344084 (PMC12965534; doi:10.1371/journal.pone.0344084)
Supplement: S1 File — (ZIP) [file pone.0344084.s001.zip › Supplementary files/Supplementary materials260218.docx]

Creating IPAH Dataset

Datasets utilized for fine-tuning and testing the Geneformer model were acquired from a publicly available database in the NCBI Gene Expression Omnibus (GEO). Specifically, we included datasets GSE169471 (Six control samples, three idiopathic pulmonary arterial hypertension [IPAH] samples), GSE210248 (three control samples, three IPAH samples), and GSE185479 (three control samples, three IPAH samples). Additionally, we incorporated a subset of the integrated Human Lung Cell Atlas (HLCA) v1.0 core dataset, specifically selecting samples annotated as lung parenchyma to augment the control dataset. Original datasets were obtained in ScanPy AnnData (h5ad) format. Quality control (QC) for GSE169471, GSE210248, and GSE185479 was conducted using the following criteria: total gene counts per cell ranging between 200 and 2500, and mitochondrial gene content below 5%. The HLCA dataset was used without further QC, as it was provided in a pre-processed format. For compatibility with Geneformer tokenization requirements, genes in the GSE210248 and GSE185479 datasets were annotated with their corresponding Ensembl IDs using the MyGene library. The GSE169471 and HLCA datasets already contained Ensembl IDs and were used directly without further modification. The prepared datasets were partitioned into three distinct groupings for downstream analysis:

1. GSE169471 only: four control samples and two IPAH samples for training, and two control samples and one IPAH sample for testing.
2. GSE169471 combined with HLCA: four control samples from GSE169471 plus 102 control samples from HLCA, and two IPAH samples for training; two control samples from GSE169471 plus five control samples from HLCA, and one IPAH sample for testing.
3. Combined datasets of GSE169471, GSE210248, GSE185479, and HLCA: For training, four control samples from GSE169471, two control samples each from GSE210248 and GSE185479, plus 102 control samples from HLCA; and two IPAH samples each from GSE169471, GSE210248, and GSE185479. For testing, two control samples from GSE169471, one control sample each from GSE210248 and GSE185479, plus five control samples from HLCA; and one IPAH sample each from GSE169471, GSE210248, and GSE185479.

Fine-tuning of Geneformer

Fine-tuning was performed to classify PAH versus control cells by leveraging the Geneformer model pre-trained on extensive transcriptional data. Specifically, we obtained the gf-12L-95M-i4096 (12-layer Transformer block, 4,096-token maximum sequence length) model from the ctheodoris/Geneformer repository on the Hugging Face Hub. For implementation, we used PyTorch along with the Hugging Face Transformers library and executed training on an H100 GPU (NVIDIA). We adapted the pre-training setup, which initially employed a masked token prediction head, by replacing it with a sequence classification head suitable for the binary classification task (PAH vs. control). The fine-tuning hyperparameters were set as follows: a learning rate of 2×10^−5^, a batch size of 64, a cosine scheduler with 100 warmup steps, and a total of eight training epochs. To mitigate overfitting, the lower four Transformer layers remained frozen during training, thereby focusing updates on the upper layers while preserving the foundational representational capacity learned during pre-training. Three separate models were created, each corresponding to one of the dataset partitions (A, B, and C) described above. These models are referred to as model A, model B, and model C, respectively. Each model was fine-tuned independently using its respective training split and evaluated on the corresponding test set to assess its classification performance.

The primer sequence used for the quantitative PCR analysis

*SOX18* Forward primer ACCGAGTTCGACCAGTACCT

Reverse primer TGTAATAGACCGCGCTGCTG

*S100A6* Forward primer TCACCATTGGCTCGAAGCTG

Reverse primer CACCTCCTGGTCCTTGTTCC

*HSP90AA1* Forward primer GCCAGTTCGGTGTTGGTTTT

Reverse primer ACTGTGAATGATCCCCCTGC

*TXNIP*　 Forward primer ACGACCCTGAAAAGGTGTACG

Reverse primer ATCCCTGCATCCAAAGCACT

*MT2A*  Forward primer CCCGCTCCCAGATGTAAAGA

Reverse primer ATAGCAAACGGTCACGGTCA

*GAPDH*  Forward primer AGCCACATCGCTCAGA

Reverse primer GCCCAATACGACCAAA

*VCAM1* Forward primer AGCACCACAGGCTCTTTTCC

Reverse primer TTGACTGTGATCGGCTTCCC

*ICAM1* Forward primer AGCTTCGTGTCCTGTATGGC

Reverse primer CTGGCACATTGGAGTCTGCT

*IL-6* Forward primer GAAAGTGGCTATGCAGTTTGAA

Reverse primer GAGGTAAGCCTACACTTTCCAAGA

*ITGB1* Forward primer CCGCGCGGAAAAGATGAAT

Reverse primer CCACAATTTGGCCCTGCTTG

Statistical information

For the knockdown efficiency assessment

*S100A6* knockdown: Control (n = 9) vs. *S100A6* KD (n = 9), P < 0.0001.

*HSP90AA1* knockdown: Control (n = 9) vs. *HSP90AA1* KD (n = 9), P < 0.0001.

*TXNIP* knockdown: Control (n = 9) vs. *TXNIP* KD (n = 9), P < 0.0001.

*MT2A* knockdown: Control (n = 9) vs. *MT2A* KD (n = 9), P < 0.0001.

For the assessment of *SOX18* mRNA expression changes following knockdown of candidate genes:

*S100A6* knockdown: Control (n = 3) vs. *S100A6* KD (n = 8), P = 0.0485.

*HSP90AA1* knockdown: Control (n = 3) vs. *HSP90AA1* KD (n = 9), P = 0.0091.

*TXNIP* knockdown: Control (n = 3) vs. *TXNIP* KD (n = 9), P = 0.0091.

*MT2A* knockdown: Control (n = 3) vs. *MT2A* KD (n = 8), P = 0.1939.

For the assessment of *VCAM1* mRNA expression changes following knockdown of candidate genes:

*S100A6* knockdown: Control (n = 4) vs. *S100A6* KD (n = 6), P = 0.1143.

*HSP90AA1* knockdown: Control (n = 4) vs. *HSP90AA1* KD (n = 6), P < 0.001.

*TXNIP* knockdown: Control (n = 4) vs. *TXNIP* KD (n = 6), P < 0.001.

For the assessment of *ICAM1* mRNA expression changes following knockdown of candidate genes:

*S100A6* knockdown: Control (n = 4) vs. *S100A6* KD (n = 6), P < 0.001.

*HSP90AA1* knockdown: Control (n = 4) vs. *HSP90AA1* KD (n = 6), P = >0.9999.

*TXNIP* knockdown: Control (n = 4) vs. *TXNIP* KD (n = 6), P = 0.0677.

For the assessment of *ITGB1* mRNA expression changes following knockdown of candidate genes:

*S100A6* knockdown: Control (n = 4) vs. *S100A6* KD (n = 6), P < 0.01.

*HSP90AA1* knockdown: Control (n = 4) vs. *HSP90AA1* KD (n = 6), P < 0.01.

*TXNIP* knockdown: Control (n = 4) vs. *TXNIP* KD (n = 6), P < 0.01.

For the assessment of *IL-6* mRNA expression changes following knockdown of candidate genes:

*S100A6* knockdown: Control (n = 4) vs. *S100A6* KD (n = 6), P = 0.3524.

*HSP90AA1* knockdown: Control (n = 4) vs. *HSP90AA1* KD (n = 6), P < 0.01.

*TXNIP* knockdown: Control (n = 4) vs. *TXNIP* KD (n = 6), P < 0.01.





**Figure S1. External validation of *SOX18* upregulation using an independent dataset.**

1. Null distribution versus actual *SOX18* change: Permutation analysis defining the natural fluctuation of gene expression. The density plot shows the distribution of Log_2_ fold changes derived from healthy donor endothelial cells (n = 1,307) in the GSE228644 dataset across 100 random split iterations. The orange shaded region represents the natural fluctuation zone, defined as the 95th percentile (log_2_ FC = 0.68). The red arrow indicates the observed Log_2_ fold change of *SOX18* in PAH versus donor cells (log_2_ FC = 0.846).
2. *SOX18* expression in endothelial cells: Violin plot comparing *SOX18* expression levels between healthy donors and PAH patients in the GSE228644 dataset (Mann-Whitney *U* test, *P* = 1.31×10⁻⁴)





**Figure S2. Effects of S100A6, HSP90AA1, and TXNIP knockdown on the expression of PAH disease-related genes.**

Relative mRNA expression levels of *VCAM1*, *ICAM1*, *ITGB1*, and *IL-6* were analyzed by RT-qPCR in control and knockdown (KD) cells. Expression levels were normalized to *GAPDH*. Graphs are presented as median with interquartile range (IQR). Statistical significance is indicated as: ** p < 0.01, ns: not significant (n = 4 for Control; n = 6 for KD).
