# Supplementary material for: PAH-former: Transfer learning for efficient discovery of pulmonary arterial hypertension-associated genes
Source: PLoS One. 2026 Mar 6;21(3):e0344084. doi: 10.1371/journal.pone.0344084 (PMC12965534; doi:10.1371/journal.pone.0344084)
Supplement: Attachment — Submitted filename: plosone_Responses to the Editor and Reviewers20260128.docx [file pone.0344084.s003.docx]

**Responses to the Editor and Reviewers**

We thank the editor and all reviewers for their insightful remarks and suggestions regarding our manuscript. We also appreciate the opportunity to revise and resubmit the manuscript.

Based on the editor and the reviewers’ comments (in **blue**), we have performed additional experiments and revised the manuscript accordingly. Our changes are highlighted in **red** in this file and in yellow in the revised manuscript file.

**Comments from the Editor**

The ms is overall interesting and informative. However, my opinion, the authors should validate the results of Sox18 comparing PAH endotheilal cells and control ECs.

**Response:**

We fully agree that validating the results in clinical samples is crucial. To address this concern and robustly validate our findings, we utilized an independent external single-cell RNA sequencing dataset (GSE228644) (1) comprising PAH and healthy donor lung samples. The analysis revealed that *SOX18* expression was significantly upregulated in PAH endothelial cells, showing an approximately 2-fold increase compared to healthy controls (Fig. S1A, B).

Furthermore, to ensure this upregulation represents a true biological signal rather than technical noise, we performed a permutation analysis, as detailed in our response to Reviewer 1. This analysis demonstrated that the observed *SOX18* upregulation significantly exceeds the natural fluctuation threshold defined by random sampling of healthy donor cells. These external validation results strongly support our original findings and confirm the pivotal role of *SOX18* in PAH.

We have included the figure below as Figure S1 in the revised manuscript. We address this in further detail in our response to Reviewer 1.

* : P < 0.05

**Comments from Reviewer #1**

Major critique- The authors need to test for other genes associated with PAH other than SOX 18. They also should have prespecified thresholds for gene regulation based on natural fluctuations and variance in normal lungs.

**Response:**

[The first half]

To address this comment, we focused on the following four genes that have been consistently reported to be highly expressed in PAH endothelial cells in previous studies: Vascular Cell Adhesion Molecule-1 (*VCAM1*), Intercellular Adhesion Molecule-1 (*ICAM1*), Interleukin-6 (*IL-6*) , and Integrin beta-1 (*ITGB1*).

*VCAM1* and *ICAM1* are proinflammatory adhesion molecules markedly elevated in the pulmonary vascular endothelium of IPAH patients and in cultured ECs from IPAH patients (2). It has been shown that the beta1 subunit, encoded by the *ITGB1* gene, is expressed at significantly higher levels compared to other beta subunits in both pulmonary artery smooth muscle cells (PASMCs) and endothelial cells (PAECs) of PAH (3). It is well established that elevated serum IL-6 levels correlate with poor prognosis in PAH patients. In murine hypoxia-induced pulmonary hypertension lungs, *IL-6* expression is markedly upregulated in pulmonary arteriolar endothelial and smooth muscle cells (4).

We performed knockdown (KD) in HPAECs targeting *S100A6*, *HSP90AA1*, and *TXNIP*, as these three genes were found to significantly upregulate *SOX18* expression in our study. Samples were collected 48 hours post-transfection, consistent with our previous experiments. We then verified whether the expression levels of the prior four PAH-associated genes were elevated in the KD group compared to the control group.

Experimental results showed a significant increase in *VCAM1* and *IL-6* levels in the *HSP90AA1* and *IL-6* KD groups compared to the control group. The extent of this rise was more pronounced for *VCAM1*. Regarding *ITGB1*, an increase was observed across all three KD groups. While *ICAM1* showed a significant increase only in the *S100A6 KD* group, the magnitude of the increase was small.

Collectively, these results indicate that the knockdown of candidates identified by PAH-former successfully induced the upregulation of other key PAH-associated genes, driving the cells toward a disease-like phenotype. However, we acknowledge that the response was variable, with some markers showing only mild or non-significant changes. This is likely because our current model predicts global state transitions but does not explicitly describe the specific molecular mechanisms leading to disease formation. We recognize this as an area for future development, and we aim to refine the algorithm to incorporate such mechanistic insights in subsequent studies

Based on the above experimental results and discussions, the paper was revised as follows.

・The qPCR results of HPAECs KD experiment were added as Fig. S2

The following description, corresponding legends and methods have been added to the text.

・The following points were added to Results (Target gene knockdown section).

“To further validate the phenotypic shift towards a PAH-like state, we additionally examined the expression of adhesion molecules (*VCAM1*, *ICAM1* and *ITGB1*) and the pro-inflammatory cytokine *IL-6*, which are known to be upregulated in PAH endothelial cells (reference). Consistent with the *SOX18* results, knockdown of *HSP90AA1* and *TXNIP* led to a significant upregulation of *VCAM1*, *ITGB1*, and *IL-6*. *S100A6* knockdown also resulted in significant increases in *ICAM1* and *ITGB1* expression (Fig. S2). These broad changes in disease-associated markers strongly support the functional relevance of the candidates identified by PAH-former.” (L.294-301)

・The following points were added to Discussion.

“The induction of a PAH-like phenotype was confirmed not only by *SOX18* upregulation but also by the increased expression of other key disease markers such as *VCAM1* and *IL-6* (Fig. S2), reinforcing the potential of these genes as drivers of the disease state. However, the extent of upregulation was variable across the tested markers. This is likely because our current model predicts global state transitions without explicitly describing the specific molecular mechanisms involved. Consequently, certain downstream pathways may not be fully activated. In future studies, we aim to refine the algorithm to better identify which specific biological pathways are mechanistically driven by the predicted gene perturbations.” (L.332-340)

“The primers used for qPCR were as follows:

*VCAM1*, AGCACCACAGGCTCTTTTCC, TTGACTGTGATCGGCTTCCC

*ICAM1*, AGCTTCGTGTCCTGTATGGC, CTGGCACATTGGAGTCTGCT

*IL-6*, GAAAGTGGCTATGCAGTTTGAA, GAGGTAAGCCTACACTTTCCAAGA

*ITGB1*, CCGCGCGGAAAAGATGAAT, CCACAATTTGGCCCTGCTTG”

(Supplementary Material)

・The following points were added to Materials and Methods (Target gene knockdown using RNA Interference section)

“*~~SOX18~~* ~~mRNA levels were evaluated for each condition at the same time.~~ The mRNA expression levels of *SOX18*, as well as *VCAM1*, *ICAM1*, *ITGB1*, and *IL-6*, were evaluated for each condition.” (L.172-173)

“**Effects of *S100A6*, *HSP90AA1*, and *TXNIP* knockdown on the expression of PAH disease-related genes.** Relative mRNA expression levels of *VCAM1*, *ICAM1*, *ITGB1*, and *IL-6* were analyzed by RT-qPCR in control and knockdown (KD) cells. Expression levels were normalized to *GAPDH*. Graphs are presented as median with interquartile range (IQR). Statistical significance is indicated as: ** p < 0.01, ns: not significant (n = 4 for Control; n = 6 for KD).” (Legend of Fig. S2)

[The second half]

To validate our findings, we utilized the independent single-cell RNA-seq dataset GSE228644. This analysis confirmed a significant upregulation of *SOX18* in PAH endothelial cells (Log_2_FC: 0.85). Furthermore, to ensure this change represents a true biological signal rather than natural variance, we performed a permutation analysis using the healthy donor cells (n = 1,307) from this dataset. We established a natural fluctuation threshold at the 95th percentile (± 0.68) based on 100 random split iterations. The observed *SOX18* upregulation significantly exceeded this noise threshold by 1.24-fold (Mann-Whitney U test, p = 1.31 × 10⁻⁴) (Fig.S1 A, B). These results provide robust external validation of our experimental data, demonstrating that *SOX18* upregulation is a distinct pathological feature that lies well beyond the range of natural fluctuations.

Based on the above experimental results and discussions, the following description, corresponding legends have been added to the text.

・The following points were added to Materials and Methods (we newly added “Estimation of natural fluctuation threshold” section.)

“To distinguish true biological signals from technical noise or natural biological variance, we performed a permutation-based analysis using the control samples from the validation dataset GSE228644. We randomly split the 1,307 healthy donor cells into two equal groups 100 times and calculated the log_2_ fold change (log_2_FC) for all genes in each iteration. From the resulting distribution of background log_2_ FC values, we defined the "natural fluctuation zone" as the range covering the 95th percentile. An observed log_2_FC in the disease group exceeding this threshold was considered a robust change beyond natural variance.” (L.186-194)

・The following points were added to Results.

“To validate our findings, we utilized an independent scRNA-seq dataset (GSE228644). This analysis confirmed a significant upregulation of *SOX18* in PAH endothelial cells compared to donors (log_2_FC = 0.85). To address the potential for natural variation, we established a natural fluctuation threshold (log_2_FC ± 0.68) based on a permutation analysis of healthy donor cells. The upregulation of *SOX18* significantly exceeded this noise threshold (1.24-fold above the threshold limit), confirming that the observed expression change represents a distinct pathological feature rather than intrinsic biological noise.“

“**External validation of *SOX18* upregulation using an independent dataset.**

(A) Null distribution versus actual *SOX18* change: Permutation analysis defining the natural fluctuation of gene expression. The density plot shows the distribution of Log_2_ fold changes derived from healthy donor endothelial cells (n = 1,307) in the GSE228644 dataset across 100 random split iterations. The orange shaded region represents the natural fluctuation zone, defined as the 95th percentile (log_2_ FC = 0.68). The red arrow indicates the observed Log_2_ fold change of *SOX18* in PAH versus donor cells (log_2_ FC = 0.846).
(B) *SOX18* expression in endothelial cells: Violin plot comparing *SOX18* expression levels between healthy donors and PAH patients in the GSE228644 dataset (Mann-Whitney *U* test, *P* = 1.31×10⁻⁴).” (Legend of Fig. S1)

The source code for PAH-former is available at the following repository <https://github.com/UTcardiology/PAH-former-analysis>. Custom scripts used for the permutation analysis and natural fluctuation threshold estimation are also available in the same repository.

Major Critique: There are many genes now identified perhaps the authors should compare vs genes with known targeted therapeutics.

**Response:**

Following this suggestion, we conducted *in silico* perturbation analyses on well-established PAH therapeutic targets, including Phosphodiesterase 5A (*PDE5A*), soluble guanylyl cyclase stimulators (*GUCY1A3*, *GUCY1B3*), endothelin receptors (*EDNRA, EDNRB*), and prostacyclin (*PTGIR*).

We tested four perturbation directions and we have attached an Excel file containing the "Shift_to_goal_end" scores for each gene, calculated via *in silico* perturbation analysis using PAH-former in this revision. We found that perturbation of these specific receptor/enzyme genes alone did not induce a statistically significant global state transition (FDR > 0.5) in our model. As mentioned in our manuscript, "Shift_to_goal_end" represents the cosine similarity shift of the cell embedding vector towards the centroid of the target state (healthy or disease) following the *in silico* perturbation of a specific gene. A higher value indicates a stronger predicted impact driving the cell toward the target state. This result likely reflects that while these targets are clinically effective for vasodilation and symptom management, modulating their individual mRNA expression levels may not be sufficient to drive a global reversion of the complex transcriptomic dysregulation characteristic of PAH in this model.

However, when we focused on the Deletion: PAH to Control direction—which simulates the therapeutic inhibition of pathogenic genes—our model successfully prioritized several previously reported driver genes of PAH, such as the *AP-1* complex, *TGF-β* (a downstream effector of PPAR-γ signaling), and *SPARC* (a target of HIF-2α) (5). In the pathway analysis of genes extracted from the "Deletion: PAH to Control" perturbation, the PID-AP1 pathway was also identified.

These findings suggest that while PAH-former may not prioritize every target genes of current vasodilators, it is highly effective at identifying upstream transcriptional regulators required to normalize the global transcriptomic state. Therefore, our strategy provides a reliable method for finding new treatment targets that address the fundamental molecular pathology of PAH.

**Summary of in silico perturbation scores for established PAH therapeutic target genes** (Extracted from supplementary table 1)

Based on the above experimental results and discussions, the paper was revised as follows.

・The following points were added to Discussion.

“Furthermore, to benchmark our model against established clinical targets, we performed *in silico* perturbation analyses on genes targeted by current PAH therapies, such as *Phosphodiesterase 5A* (*PDE5A*), *Guanylate Cyclase 1 Soluble Subunit Alpha 3* (*GUCY1A3*), *Endothelin Receptor Type A* (*EDNRA*), and *Prostaglandin I2 Receptor* (*PTGIR*). Interestingly, perturbation of these individual genes did not induce a significant global shift in cell embedding towards the healthy state. While these targets are clinically effective for vasodilation and symptom management, modulating their expression alone may not be sufficient to reverse the complex, global transcriptomic dysregulation characteristic of the PAH endothelial phenotype. In contrast, when we focused on the *in silico* deletion analysis simulating the reversion from the PAH state to the control phenotype—which simulates the therapeutic inhibition of pathogenic genes—our model successfully prioritized major upstream regulators and signaling nodes, such as the Activator Protein 1 (*AP-1*) complex, Transforming Growth Factor Beta (*TGF-β*), and Secreted Protein Acidic and Cysteine Rich (*SPARC*) which are well-known key factors involved in the pathophysiology of PAH (5). This suggests that PAH-former is particularly effective at identifying fundamental drivers required to normalize the global cellular state.” (L.347-363)

Major Critique: the figures are not of high quality; some of the data is illegible.

**Response:**

We addressed your comment.

**Comments from Reviewer #2**

Major Comments: For the target gene knockdown analysis section, the authors mentioned picking four genes for further validation. It is not clear what the rationale was behind choosing these genes. Is it data-driven or biology driven? This needs to be clarified or cleared up in the manuscript.

**Response:**

The identification of the four genes for validation was guided by a combined approach that prioritized data-driven predictions while incorporating biological context and experimental feasibility. First, we identified top-ranked genes with high control to PAH shift scores, which is previously mentioned as “Shift_to_goal_end”. Next, we carefully examined each of these top candidates' biological functions one by one. In consultation with the PAH experts among the co-authors, we carefully chose genes with functional traits that clearly suggested a possible connection to the pathophysiology of PAH, even though they had not previously been directly reported in the previous literature. Finally, we confirmed that these candidates showed detectable basal expression in HPAECs based on public transcriptomic data (GSE228644) to ensure experimental feasibility.

・The following points were added to Materials and Methods (Target Gene Knockdown using RNA Interference section). The term ‘HPAECs’ is spelled out at its first mention.

**“**To choose targets for experimental validation, we used a multi-step procedure. Top-ranked genes discovered by Control-to-PAH shift scores were manually curated, which included consultation with domain experts, to highlight physiologically viable but previously unknown candidates. Candidates were further screened for basal expression in human pulmonary artery endothelial cells (HPAECs) using the GSE228644 dataset (reference), ensuring the feasibility of subsequent validation experiments.**”** (L.147-153)

Major comments: Also, while the authors performed experiments associated with both in silico deletion and overexpression analysis using the PAH-former, the candidate genes were picked from the 134 gene list coming out of the in silico deletion analysis only. What is the reason behind it? Did the authors explore any candidates from the overexpression analysis?

**Response:**

Although our primary validation focused on the deletion analysis, we did indeed explore the candidate genes prioritized by the *in silico* overexpression analysis. This exploration yielded several biologically compelling candidates, such as Ephrin-B2 (*EFNB2*), a key determinant of arterial endothelial identity (6), and Repulsive Guidance Molecule BMP Co-Receptor A (*RGMA*), a co-receptor for the BMP signaling pathway. These findings support the validity of our model in both directions. To facilitate rapid experimental validation of the model's predictions, we prioritized the knockdown approach, which offers a more efficient workflow for testing multiple targets compared to overexpression. Consequently, we focused on deletion candidates in this study. We have added a discussion regarding these overexpression candidates to the revised manuscript to highlight the model's predictive capability and outline our future directions.

・The following points were added to Discussion (just before Limitation section).

“Our *in silico* overexpression analysis also identified promising candidates, such as Ephrin-B2 (*EFNB2*) and Repulsive Guidance Molecule BMP Co-Receptor A (*RGMA*), which are known to play critical roles in vascular homeostasis (reference) and BMP signaling. Although we prioritized the experimental validation of knockdown targets in this study, the identification of these established markers suggests that our model captures bidirectional regulatory dynamics. Validation of these overexpression candidates is planned for future investigations.” (L.375-381)

Major comments: The data partitions for fine-tuning experiments seem to be a bit off. Specifically for model B, looks like the authors used a total of 106 controls + 2 IPAH samples for training while using 1 IPAH sample + 7 controls. Did the authors try using similar IPAH:controls ratio for both training and testing partitions? Also, what are the cell counts in these partitions? It might be relevant to include those in the manuscript.

**Response:**

We clarify the exact cell counts and sampling strategy below.

For the training datasets:

- Model A was trained on 6,514 PAH cells and 5,106 control cells.
- Model B extends the Model A dataset by adding 10,000 control cells randomly sampled from 102 control samples. As a result, the training set for Model B consists of 6,514 PAH cells and 15,106 control cells.
- Model C further augments the Model B dataset by incorporating eight additional PAH samples from GSE185479 (7) and GSE210248 (1), from which 6,000 IPAH cells were randomly sampled. The final training dataset for Model C therefore contains 12,514 PAH cells and 15,106 control cells.

For the test dataset, the same dataset was used for evaluation across Models A–C. This test set was constructed from samples in GSE169471 that were not included in the training data of Model A (1 PAH sample and 2 control samples), and consists of 1,839 PAH cells and 2,780 control cells.

Regarding the IPAH-to-control ratio, while it was not possible to exactly match the ratios across all training and test partitions, the ratios fall within a range of approximately 1 : 0.8 to 1 : 2.3. We believe this range does not represent a severe class imbalance and is unlikely to introduce a major bias in model training or evaluation.

・The following points were added to Materials and Methods (Model evaluation section).

“For consistent evaluation, a common test set was constructed from samples in GSE169471 that were not included in the training data (1 PAH sample and 2 control samples), consisting of 1,839 PAH cells and 2,780 control cells.” (L.103-106)

“Model A: Fine-tuned using only the GSE169471 dataset (6,514 PAH cells and 5,106 control cells).

Model B: Fine-tuned using the GSE169471 dataset combined with ~~a large set of control samples~~ 10,000 control cells randomly sampled from the Human Lung Cell Atlas (HLCA) (6,514 PAH cells and 15,106 control cells).

Model C: Fine-tuned using a combined dataset comprising all three PAH-related datasets (GSE169471, GSE210248, and GSE185479) with 6,000 additional PAH cells randomly sampled, plus the control samples from the HLCA (12,514 PAH cells and 15,106 control cells). (L.109-117)

Major comments: In the results section, the authors compared the disease related genes identified by PAH-former with DEGs from the previous article. In the 690 genes identified specifically by the PAH-former, were there any functional terms (pathways or processes) that the relevant to PAH or pulmonary disorders in general? If that is the case, it could be another good justification for using such complex models over the traditional DEG analysis.

**Response:**

As shown in Figure 4B, there was minimal overlap (only two genes) between the genes identified by PAH-former and those identified by conventional DEG analysis. Therefore, the enrichment analysis of the PAH-former candidates presented in the Results section (Figure 3B-E) essentially represents the functional profile of the genes specifically identified by our model. As noted in the text, these unique candidates were significantly enriched in pathways central to PAH pathogenesis, such as TNF-α/NF-κB signaling, inflammatory response, oxidative stress response, and VEGFA/VEGFR2 signaling (Results section). We think this ability to capture biologically relevant pathways that traditional DEG analysis missed is a key justification for using our deep learning approach. In the revised manuscript, we emphasized this point to clarify the advantage of PAH-former over conventional methods.

・The following points were added to Results.

“This demonstrates that PAH-former effectively found functionally significant disease pathways that were missed by conventional DEG analysis, highlighting the importance of using a context-aware deep learning model.” (L.263-266)

Minor comments:

- Looks like the authors used the short form ‘HPAECs’ before it was defined for the first time in the manuscript (the full-form was mentioned in a later section of the manuscript). This needs to be fixed.

**Response**

We addressed your comment. We replaced 'human pulmonary endothelial cells (HPAECs)' with 'HPAECs' in the Target Gene Knockdown' section, as it was not the first mention. (L.282)

We have spelled out the full term at its first appearance in the manuscript. (L.151)

- The word ‘which’ was misspelled (‘whcih’) in line 246 in the ‘Target Gene Knockdown’ section. Please correct this.

**Response**

We addressed your comment. The misspelled ‘which’ is now in L.272.

- From a reproducibility perspective, making the scripts used for fine-tuning the Geneformer model can be very useful. The authors should consider sharing their work.

**Response**

We have made the source code publicly available on GitHub <https://github.com/UTcardiology/PAH-former-analysis>.

Finally, we added the following references during the revision. We have adjusted the reference numbering in the revised manuscript to match the order of citation.

1.	Crnkovic S, Valzano F, Fließer E, Gindlhuber J, Thekkekara Puthenparampil H, Basil M, et al. Single-cell transcriptomics reveals skewed cellular communication and phenotypic shift in pulmonary artery remodeling. JCI Insight [Internet]. 2022 Oct 24 [cited 2026 Jan 25];7(20). Available from: http://dx.doi.org/10.1172/jci.insight.153471

2.	Le Hiress M, Tu L, Ricard N, Phan C, Thuillet R, Fadel E, et al. Proinflammatory signature of the dysfunctional endothelium in pulmonary hypertension. Role of the macrophage migration inhibitory factor/CD74 complex. Am J Respir Crit Care Med. 2015 Oct 15;192(8):983–97.

3.	Lemay S-E, Montesinos MS, Grobs Y, Yokokawa T, Shimauchi T, Mougin M, et al. Exploring integrin α5β1 as a potential therapeutic target for pulmonary arterial hypertension: Insights from comprehensive multicenter preclinical studies. Circulation. 2025 Apr 22;151(16):1162–83.

4.	Hashimoto-Kataoka T, Hosen N, Sonobe T, Arita Y, Yasui T, Masaki T, et al. Interleukin-6/interleukin-21 signaling axis is critical in the pathogenesis of pulmonary arterial hypertension. Proc Natl Acad Sci U S A. 2015 May 19;112(20):E2677-86.

5.	Yang L, Wan N, Gong F, Wang X, Feng L, Liu G. Transcription factors and potential therapeutic targets for pulmonary hypertension. Front Cell Dev Biol. 2023 Mar 17;11:1132060.

6.	Aoyagi Y, Schwartz AW, Li Z, Bai H, Gonzalez L, Lazcano Etchebarne C, et al. Changes in vascular identity during vascular remodeling. JVS Vasc Sci. 2025 Feb 25;6(100282):100282.

7.	Asosingh K, Comhair S, Mavrakis L, Xu W, Horton D, Taylor I, et al. Single-cell transcriptomic profile of human pulmonary artery endothelial cells in health and pulmonary arterial hypertension. Sci Rep. 2021 July 19;11(1):14714.
